# Effects of Soil Amendments on Heavy Metal Immobilization and Accumulation by Maize Grown in a Multiple-Metal-Contaminated Soil and Their Potential for Safe Crop Production

**DOI:** 10.3390/toxics8040102

**Published:** 2020-11-11

**Authors:** Fayuan Wang, Shuqi Zhang, Peng Cheng, Shuwu Zhang, Yuhuan Sun

**Affiliations:** 1 College of Environment and Safety Engineering, Qingdao University of Science and Technology, Qingdao 266042, China; wangfayuan@qust.edu.cn (F.W.); zsq9629@163.com (S.Z.); cp1995a@163.com (P.C.); zhangshuwu@126.com (S.Z.); 2 Key Laboratory of Soil Resources and Environment in Qianbei of Guizhou Province, Zunyi Normal University, Zunyi 563002, China

**Keywords:** soil remediation, heavy metal immobilization, soil pollution, food safety

## Abstract

Soil amendments have been proposed for immobilizing metallic contaminants, thus reducing their uptake by plants. For the safe production of crops in contaminated soil, there is a need to select suitable amendments that can mitigate heavy metal uptake and enhance crop yield. The present experiment compared the effects of three amendments, hydroxyapatite (HAP), organic manure (OM), and biochar (BC), on plant growth and heavy metal accumulation by maize in an acidic soil contaminated with Cd, Pb, and Zn, and their potential for safe crop production. Toxicity characteristic leaching procedure (TCLP) tests, energy dispersive X-ray spectroscopy (EDS) analysis, and X-ray diffraction (XRD) analysis were used to evaluate the effectiveness and mechanisms of heavy metal immobilization by the amendments. The results showed that shoot and root biomass was significantly increased by HAP and 1% OM, with an order of 1% HAP > 0.1% HAP > 1% OM, but not changed by 0.1% OM and BC (0.1% and 1%). HAP significantly decreased Cd, Pb, and Zn concentrations in both shoots and roots, and the effects were more pronounced at the higher doses. OM decreased the shoot Cd and Pb concentrations and root Zn concentrations, but only 1% OM decreased the shoot Zn and root Pb concentrations. BC decreased the shoot Cd and Pb concentrations, but decreased the shoot Zn and root Pb concentrations only at 1%. HAP decreased the translocation factors (TFs) of Cd, Pb, and Zn (except at the 0.1% dose). OM and BC decreased the TFs of Cd and Zn, respectively, at the 1% dose but showed no significant effects in other cases. Overall, plant P, K, Fe, and Cu nutrition was improved by HAP and 1% OM, but not by 0.1 OM and BC. Soil pH was significantly increased by HAP, 1% OM, and 1% BC, following an order of 1% HAP > 1% OM > 0.1% HAP > 1% BC. The TCLP levels for Cd, Pb, and Zn were significantly reduced by HAP, which can be partly attributed to its liming effects and the formation of sparingly soluble Cd-, Pb-, and Zn-P-containing minerals in the HAP-amended soils. To some extent, all the amendments positively influenced plant and soil traits, but HAP was the optimal one for stabilizing heavy metals, reducing heavy metal uptake, and promoting plant growth in the contaminated soil, suggesting its potential for safe crop production.

## 1. Introduction

Soil contamination with heavy metals represents a global environmental issue with adverse consequences for the environment and human health. It is estimated that, globally, more than 20 million hm^2^ of land is contaminated with heavy metal(loid)s [1]. Particularly, heavy metals can enter agricultural soils via various pathways, such as atmospheric deposition, sewage irrigation, and applications of livestock manures and agricultural chemicals [2,3]. They can be taken up by crops and accumulated in edible parts, and they further pose potential health risks for humans and animals via the food chain [4,5]. To remedy heavy metal-polluted soils, various in situ and ex situ remediation techniques have been developed in recent decades [1,6].

Soil amendments have been proposed to remediate soils contaminated with various heavy metals [7,8,9]. The commonly used amendments include natural organic materials, such as compost, sewage sludge, biochar, humic substances, and plant extracts and exudates; inorganic materials such as lime and phosphate; and chemical chelators such as ethylenediaminetetraacetic acid (EDTA), diethylenetriaminepentaacetic acid (DTPA), ethylenediaminedisuccinic acid (EDDS), malic acid, and citric acid. Many amendments can immobilize heavy metals through precipitation, complexation, ion exchange, and adsorption, but they have different specific characteristics and dominant remediation mechanisms [10,11,12,13,14,15]. In addition, when amendments are used in the remediation of contaminated agricultural fields, their effects on crop growth and heavy metal accumulation should be taken into account, considering the potential human health risks of heavy metals in the food chain [16].

Among the commonly used amendments, hydroxyapatite (HAP) is effective for dealing with heavy metal-contaminated soil. HAP has a unique hydroxyl group that enhances soil pH, diminishing the solubility of heavy metals, such as Cd and Pb, by forming chelate or precipitate substances to change heavy metal bioavailability and reduce phytotoxicity [17,18,19,20]. HAP can not only directly immobilize heavy metals, leading to reduced heavy metal toxicity, but also improve plant growth and biomass, and reduce heavy metal content in plant tissues [21,22]. Meanwhile, HAP can be considered an inorganic P fertilizer, providing P nutrition for plants. It is necessary to study its remediation effects for safe crop production in contaminated soil.

Biochar (BC) is an amendment with an excellent adsorption capacity obtained from organic materials under high-temperature anaerobic conditions [23]. The adsorption capacity of biochar for heavy metals is mainly due to the presence of many oxygen-containing functional groups and a large specific surface area [24], which can reduce the migration capacity of heavy metals in soil, and improve the physical and chemical properties of contaminated soil [25,26], including the pH, cation exchange capacity (CEC), and soil water capacity [27,28,29]. In recent years, biochar has been widely used in soil remediation [14,15,30]. Biochar can adsorb soil heavy metals, reduce plant toxicity while increasing plant biomass [31], improve plant water-use efficiency [32], and decrease heavy metal concentrations in plants [33], indicating potential for safer crop production. However, the effects vary with soil properties, plant species, and metal contaminants, which should be clarified before any realistic application.

As a common organic fertilizer, organic manure (OM) can improve soil quality and enhance crop production, with the advantages of low cost and abundant sources. In addition, manure can also reduce heavy metal toxicity through complexation and adsorption [34]. Organic materials in manure contain functional groups such as carboxyl groups and hydroxyl groups, which combine with heavy metals to form organic–metal complexes, altering the migration and availability of heavy metals [35,36]. In a Cd-contaminated soil treated with organic manure, *Bidens tripartite* biomass increased, while the Cd content in plants decreased [37]. However, in a soil contaminated with Cd and Zn, pig manure increased heavy metal phytoextraction by *Streptomyces pactum* [38]. In our previous studies, cattle manure generally decreased Pb and Cd concentrations in tobacco plant tissues, but increased the total uptake of Pb and Cd, due to the greater plant biomass [39,40]. Thus, the effects of OM on heavy metal uptake by plants may vary with the manure source, the plant species, the heavy metal content and availability in the soil, and other soil conditions, which should be clarified for crop production in a realistically contaminated soil.

In one of our remediation projects for an acidic soil contaminated with multiple heavy metals, soil amendments accompanying crop production are planned to be implemented. However, it is not known which amendment is optimal for the target soil and the crop. Based on the above context, three amendments with different remediation mechanisms, i.e., HAP, OM, and BC, were tested using greenhouse experiments prior to field applications. We aimed to compare the effects of these amendments on heavy metal immobilization, plant growth, and heavy metal accumulation, and to select appropriate soil amendments for safe maize production in the contaminated soil.

## 2. Materials and Methods

### 2.1. Soil

The test soil was collected from the surface layer (0–20 cm depth) of an abandoned rice field (25°10′35.15′′ N, 113°64′85.97′′ S), located in Dongtang town, Renhua County, Shaoguan, Guangdong Province, China. This field was polluted due to the nearby Pb/Zn smelter 1.5 km away in the northeast. About 200 kg of fresh soil was sampled and completely mixed. The soil was air-dried, crushed, and then sieved through a 2 mm mesh. The physicochemical properties of the test soil were determined based on the methods described by Lu [41] (Table 1).

According to China’s soil environmental quality for agricultural land standard (GB15618-2018), the concentrations of Cd and Pb are higher than the soil intervention value, and the Zn concentration is seven times higher than the soil screening value (Table 1). Thus, the soil can be regarded as contaminated by Cd, Pb, and Zn.

### 2.2. Plants and Soil Amendments

The seeds of maize (*Zea mays* L. var. Wannuoyihao) were surface sterilized by steeping them in a 2% NaClO solution for 15 min and then washed three times with deionized water.

Cow dung-based manure was kindly provided by Luoyang Wonong Agricultural Technology Co., Ltd. (Luoyang, China). The manure (pH 8.58) contained ~60% organic matter, ~30% water content, and ~5% total contents of N, P, and K. Prior to use, the manure was sieved using a 2 mm sieve.

Hydroxyapatite (HAP) was purchased from Shanghai Hualan Chemical Technology Co., Ltd. (Shanghai, China). HAP is an ultra-fine powder with a pH of 8.10, Ca_10_(PO_4_)_6_OH_2_ content ≥ 99.5%, total heavy metal content ≤ 1 mg/kg, and average particle size ≤ 40 μm.

Biochar was purchased from Qingdao Biochar Environmental Bioengineering Co. Ltd. (Qingdao, China). It was obtained by heating pine shoots under a 700 °C oxygen-free environment for 2 h, with the following properties: pH, 9.64; average specific surface area, 139.4 m^2^/g; C, 86.15%; H, 3.17%; N, 0.29%; S, 0.51%; O, 7.36%; zeta potential, −26 mV; and average particle size, 9.5 μm. It was used to remediate Cr(VI)-contaminated soil in our recent study [42]. The SEM images and FTIR spectra of the biochar are shown in Figure S1 (see Supplementary Materials). The ground biochar was sieved using a 2 mm sieve for use.

### 2.3. Experimental Design and Procedure

The pot experiment included three amendment treatments, i.e., hydroxyapatite (HAP), organic manure (OM), and biochar (BC). Each amendment was applied individually. Two application doses, 0.1% and 1% (*w*/*w*), were designed to represent low and high doses, respectively. A control treatment receiving no amendments was also included. Each treatment included six replicates. A proper amount of each amendment was mixed thoroughly into the soil to achieve the target concentrations. Thereafter, 1200 g of soil with or without amendments was placed into each pot (1.4 L volume; top diameter, 15.5 cm; bottom diameter, 10.1 cm). Ten surface-sterilized seeds were grown in each pot, and nine seedlings were retained one week after seeding emergence. The seedlings were placed in the chamber, with a temperature of 20–25 °C, a humidity of 40–60%, and a light/darkness photoperiod of 16/8 h (light intensity, 10,000 Lux). Deionized water was irrigated to maintain a normal soil water content (about 20%) for plant requirements.

### 2.4. Sample Analysis

After cultivation for 60 days, plant shoot and root samples were harvested separately and immediately washed with tap water and deionized water. The fresh samples were oven-dried at 70 °C for 24 h and then weighed as dry weight.

Dry plant samples (about 0.5 g) were added to the digestion tube for further HNO_3_ digestion with a Graphite Digestion Instrument (SH220N, Shandong Hanon Instruments Co. Ltd., Jinan, China). The contents of metals (Cd, Pb, Zn, K, Fe, Mn, and Cu) and P were determined with ICP-MS (Agilent 7700, Palo Alto, CA, USA).

The soil from each pot was mixed thoroughly and sampled for the analysis of metal availability and soil pH. The available forms of Cd, Pb, and Zn in the soil were evaluated using the TCLP test [43]. The available Fe, Mn, and Cu in the soil were evaluated using DTPA solution (0.005 M DTPA, 0.1 M triethanolamine, 0.01 M CaCl_2_, pH 7.3) [44]. The metal concentrations in the extracts were determined using ICP-MS. A soil solution (soil/water, 1:2.5, *w*/*v*) was used to assay the soil pH using a pH meter (pHS-3C, Sanxin, Shanghai, China).

HAP was chosen manually from air-dried soil under microscopy for analysis using an X-ray diffractometer (Rigaku D-Max-2500/PC, Rigaku Industrial Corp., Tokyo, Japan). Samples were scanned from 10 to 90 º with CuKα radiation at 40 kV and 150 mA. EDS analysis was performed using a Bruker XFlash 6130 energy dispersive spectrometer (Bruker Corp., Karlsruhe, Germany).

### 2.5. Data Analysis

The amount of heavy metal uptake by plants can be calculated from the heavy metal content in the shoot/root multiplied by the shoot/root dry weight [38]. Meanwhile, the translocation factor (TF) refers to the ratio of the heavy metal concentration in the shoot to that in the root (Equation (1)).

TF = *C*_shoot_*/C*_root_ (1)

*C*_shoot_ and *C*_root_ are the heavy metal contents (mg/kg) in the shoot and root, respectively.

Data were analyzed using SPSS 24.0 (IBM SPSS, Chicago, IL, USA) to obtain the significance and variance. Duncan’s multiple range test was used for a two-way ANOVA analysis (*p* < 0.05) to compare the effects of interactions between amendments and their dosages. Pearson correlation coefficients were calculated to determine the correlations among different parameters.

## 3. Results

### 3.1. Soil pH

**Figure 1:**
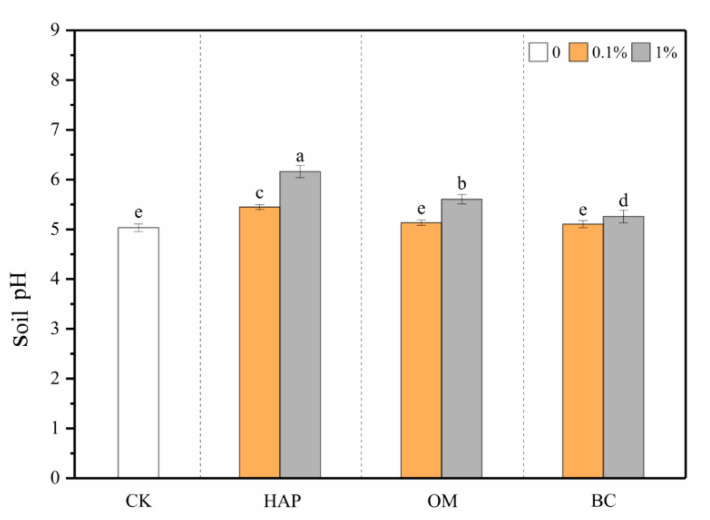
Soil pH (means ± SD, *n* = 6) after plant harvest. CK represents the control treatment. Different letters above the bars indicate significant differences among all means according to a one-way ANOVA followed by Duncan’s test (*p* < 0.05). Two-way ANOVA results are shown in Table 2.

Compared to the treatments without amendments, the three amendments all increased soil pH when applied at the 1% dose, following an order of HAP > OM > BC (Figure 1). For example, in the soil receiving 0.1% and 1% HAP, the pH value reached 5.45 and 6.16, respectively, significantly higher than the pH value of 5.03 in the soil without amendments. When applied at the 0.1% dose, only HAP enhanced the soil pH, while OM and BC had no significant impacts. The two-way ANOVA results show that there were significant interactive effects between the amendment type and dose on the soil pH (Table 2).

### 3.2. TCLP-Cd, -Pb, and -Zn in Soil

**Figure 2:**
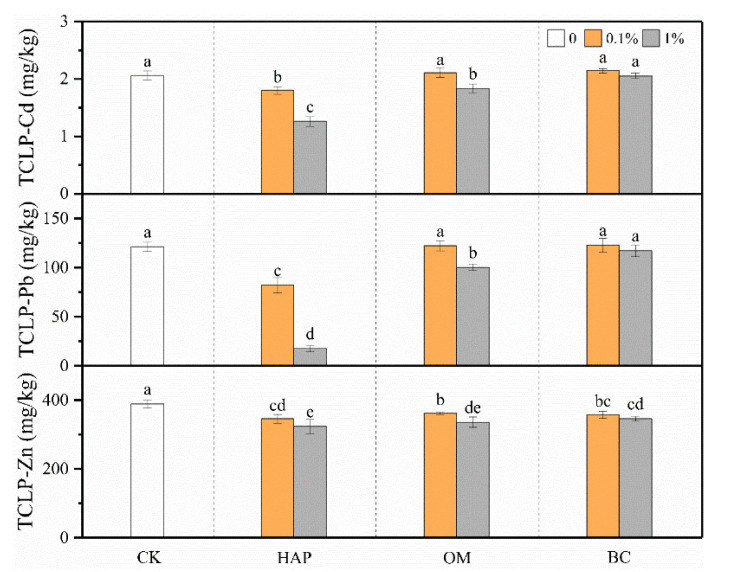
TCLP-Cd, -Pb, and -Zn concentrations (means ± SD, *n* = 6) in soil after plant harvest. CK represents the control treatment. Different letters above the bars indicate significant differences among all means according to a one-way ANOVA followed by Duncan’s test (*p* < 0.05). Two-way ANOVA results are shown in Table 2.

Compared to the treatments without amendments, HAP significantly decreased TCLP-Cd, -Pb, and -Zn concentrations in the soil, and the effects were more pronounced at the higher dose (Figure 2). For example, when 1% HAP was applied, the TCLP-Cd, -Pb, and -Zn concentrations decreased by 39%, 86%, and 17%, respectively, compared to the treatment without amendments. Even when 0.1% HAP was applied, the deceases still reached 12%, 32%, and 11%, respectively. OM decreased the TCLP-Zn concentrations, but only 1% OM decreased the TCLP-Cd and -Pb concentrations. BC reduced only the TCLP-Zn concentration, but did not significantly influence TCLP-Cd and -Pb. The two-way ANOVA results show that there were significant interactive effects between the amendment type and dose on the TCLP-Cd, -Pb, and -Zn concentrations (Table 2).

### 3.3. Plant Biomass

**Figure 3:**
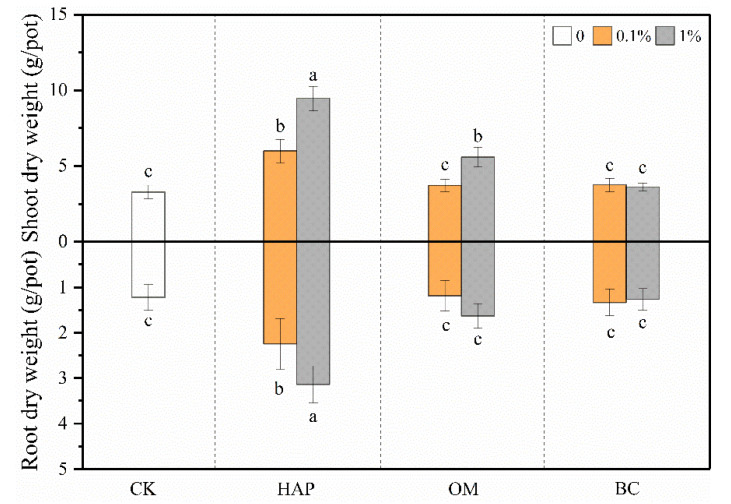
Dry weights (means ± SD, *n* = 6) of maize shoots and roots. CK represents the control treatment. Different letters above or below the bars indicate significant differences among all means according to a one-way ANOVA followed by Duncan’s test (*p* < 0.05). Two-way ANOVA results are shown in Table 2.

Compared to the control treatment, HAP increased the shoot and root biomass by 188% and 157%, respectively, at the 1% dose and by 83% and 84%, respectively, at the 0.1% dose. OM at the 1% dose increased the shoot and root biomass by 70% and 34%, respectively, but had no significant effects at the 0.1% dose. Either 0.1% or 1% BC did not significantly change plant biomass (Figure 3, Table 2). The two-way ANOVA results show significant interactive effects on the shoot and root biomass between the amendment type and dose (Table 2).

### 3.4. Heavy Metal Concentrations, Uptake, and TF in Plants

**Figure 4:**
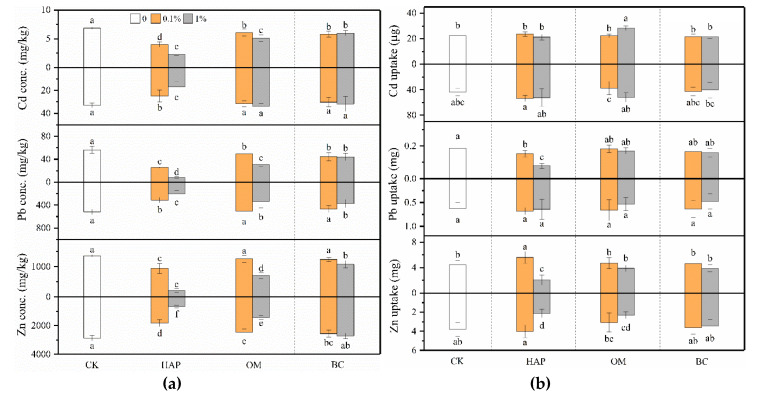
Concentrations (**a**) and uptake (**b**) (means ± SD, *n* = 6) of Cd, Pb, and Zn in maize shoots (above X-axis) and roots (below X-axis). CK represents the control treatment. Different letters above or below the bars indicate significant differences among all means according to a one-way ANOVA followed by Duncan’s test (*p* < 0.05). Two-way ANOVA results are shown in Table 2.

Compared to the treatments without amendments, HAP significantly decreased the Cd, Pb, and Zn concentrations in both the shoots and roots, and the effects were more pronounced at the higher dose (Figure 4a). For example, when 1% HAP was applied, shoot Cd, Pb, and Zn concentrations decreased by 67%, 85%, and 84%, respectively, compared to the treatment without amendments. Even when 0.1% HAP was applied, the deceases still reached 42%, 55%, and 31%, respectively. OM decreased the Cd and Pb concentrations in the shoots and Zn concentrations in the roots, but only 1% OM decreased the shoot Zn and root Pb concentrations. BC decreased the shoot Cd and Pb concentrations, but decreased the shoot Zn and root Pb concentrations only at 1%. The two-way ANOVA results showed that there were significant interactive effects between the amendment type and dose on the shoot Cd, Pb, and Zn concentrations and root Cd and Zn concentrations (Table 2).

HAP decreased shoot Pb uptake, but did not significantly change shoot Cd uptake, and root Cd and Pb uptake (Figure 4b). HAP decreased shoot and root Zn uptake at 1% but increased shoot Zn uptake at 0.1%. OM increased shoot Cd uptake and decreased root Zn uptake at 1%, but showed no significant effects in other cases. Either 0.1% or 1% BC did not change Cd, Pb, and Zn uptake in shoots and roots. The two-way ANOVA results show that there were significant interactive effects between the amendment type and dose on shoot Cd, Pb, and Zn uptake and root Zn uptake (Table 2).

**Figure 5:**
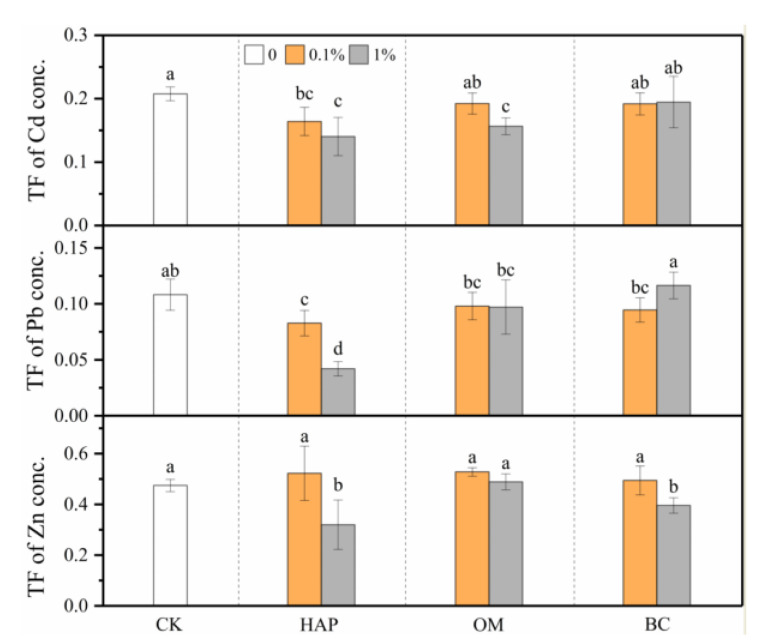
TF of Cd, Pb, and Zn (means ± SD, *n* = 6) in maize plants. CK represents the control treatment. Different letters above the bars indicate significant differences among all means according to a one-way ANOVA followed by Duncan’s test (*p* < 0.05). Two-way ANOVA results are shown in Table 2.

Overall, HAP deceased the TF of Cd, Pb, and Zn (except at the 0.1% dose) (Figure 5). OM and BC decreased the TF of Cd and Zn, respectively, at the 1% dose but showed no significant effects in other cases.

### 3.5. Plant Mineral Nutrition

**Figure 6:**
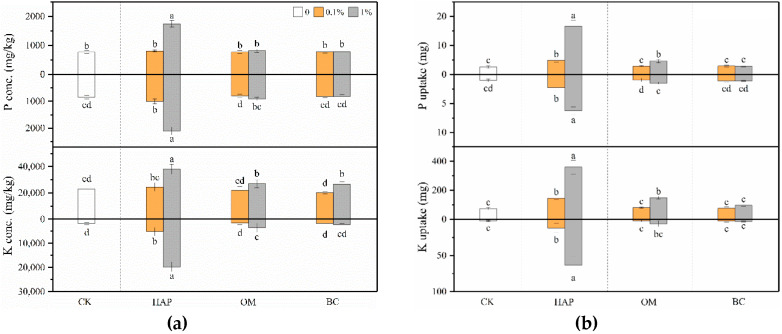
Concentrations (**a**) and uptake (**b**) (means ± SD, *n* = 6) of P and K in maize shoots (above X-axis) and roots (below X-axis). CK represents the control treatment. Different letters above or below the bars indicate significant differences among all means according to a one-way ANOVA followed by Duncan’s test (*p* < 0.05). Two-way ANOVA results are shown in Table S1.

Compared to the treatments without amendments, 1% HAP significantly increased the P and K concentrations and uptake in both shoots and roots (Figure 6). HAP at the 0.1% dose did not significantly increase shoot P and K concentrations, but enhanced P and K uptake in both shoots and roots. OM benefited plant P and K nutrition only at 1%, but exhibited no significant impacts at 0.1%. In most cases, BC produced no promoting effects, but 1% BC improved the shoot K concentration.

**Figure 7:**
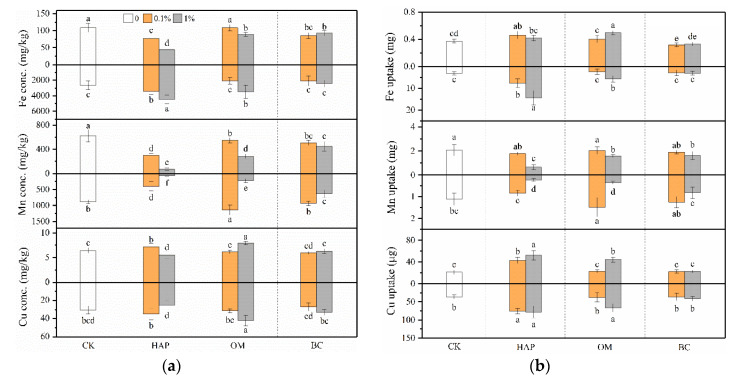
Concentrations (**a**) and uptake (**b**) (means ± SD, *n* = 6) of Fe, Mn, and Cu in maize shoots (above X-axis) and roots (below X-axis). CK represents the control treatment. Different letters above or below the bars indicate significant differences among all means according to a one-way ANOVA followed by Duncan’s test (*p* < 0.05). Two-way ANOVA results are shown in Table S1.

HAP at both doses and 1% OM increased Fe and Cu uptake in both shoots and roots (Figure 7). In most cases, BC did not significantly influence plant Fe and Cu uptake. HAP, OM, and BC all decreased the shoot Mn concentrations at both doses, and decreased shoot and root Mn uptake at the 1% dose.

### 3.6. EDS and XRD Analyses

**Figure 8:**
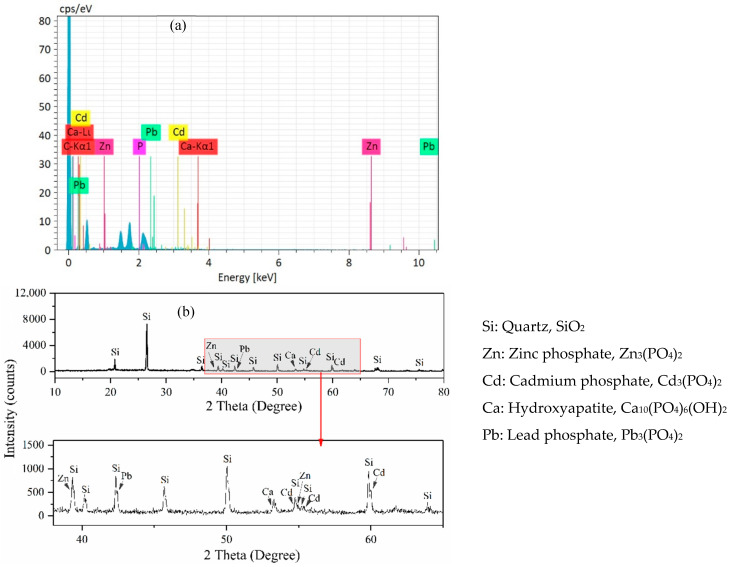
EDS image (**a**) and XRD pattern (**b**) of hydroxyapatite (HAP) in the soil after plant harvest.

EDS analysis of HAP isolated from the soil after plant harvest is presented in Figure 8a. The characteristic peaks of Cd, Pb, and Zn were identified by the EDS analysis (Figure 8a), confirming that these metals occurred on the surfaces of the HAP.

The XRD patterns of the HAP after plant harvest (Figure 8b) show the presence of characteristic peaks of several Cd-, Pb-, and Zn- phosphates, confirming the formation of sparingly soluble P-containing minerals with these metals.

### 3.7. Correlations between Soil and Plant Traits

Pearson correlation analyses showed that the soil pH was positively correlated with the shoot and root biomass (*p* < 0.01) but negatively so with the concentrations of Cd, Pb, and Zn in shoots and roots (*p* < 0.01) and the TF of Cd, Pb, and Zn (*p* < 0.01) (Table 3). Furthermore, TCLP-extractable metals were negatively correlated with the shoot and root biomass (*p* < 0.01) but positively so with their corresponding concentrations in plants (*p* < 0.01) and TF (except Zn) (*p* < 0.01).

## 4. Discussion

In the present study, we compared the effects of three amendments on heavy metal immobilization and heavy metal accumulation by plants in a multiple-heavy-metal-contaminated soil. Overall, the three amendments decreased heavy metal bioavailability and concentrations in plant shoots, and HAP showed the most pronounced effects. At the same time, plant biomass and nutrition were improved by HAP and OM, and soil acidity was mitigated by the three amendments. These results suggest that these soil amendments have promising potential in the remediation of contaminated soil and the safe production of crops therein.

Phosphates, manures, and biochars can all reduce heavy metal solubility through diverse immobilization mechanisms such as precipitation, complexation, ion exchange, and adsorption [10,11,12,13,14,15]. We found that the amendments not only decreased heavy metal bioavailability but also mitigated heavy metal contents in plants, but the effectiveness varied with amendment type and dose. Overall, HAP was the most effective amendment, and even the 0.1% dose had better mitigating effects than OM and BC (Figure 2 and Figure 4). These facts imply that they have different specific characteristics and remediation mechanisms. Generally, complexation and adsorption, instead of precipitation, may dominate in the immobilization of heavy metals by manure and biochar [6,15]. However, the immobilization of metals by HAP is mainly due to the formation of phosphate precipitates with much lower solubility and bioaccessibility [11,45,46]. P-containing amendments generally decrease Pb mobility by leading to the precipitation of pyromorphite-type minerals (Pb_5_(PO_4_)_3_X; X = F, Cl, Br, or OH) [47]. For example, HAP can convert soil Pb to pyromorphite (Pb_5_(PO_4_)_3_Cl) [19]. Co-precipitation is considered the main process for immobilizing Cd with HAP by forming (Cd_x_, Ca_10−x_)(PO_4_)_6_(OH)_2_ [48,49]. In our present study, XRD analyses confirmed the formation of some insoluble P-containing minerals on the surfaces of HAP, such as Cd_3_(PO_4_)_2_, Pb_3_(PO_4_)_2_, and Zn_3_(PO_4_)_2_ (Figure 8b), which partly explains the lower TCLP-Cd, -Pb, and -Zn concentrations in the soil amended with HAP.

The liming effect is among the most common immobilization mechanisms. Soil pH generally influences the mobility and bioavailability of metallic contaminants and their absorption by plants [50,51]. A high soil pH generally facilitates the immobilization of Cd and Pb, leading to lower phytoavailability [52]. Our results show that HAP at both doses and OM and BC at the 1% dose all increased the soil pH (Figure 1), and the soil pH correlated positively with the plant biomass but negatively (*p* < 0.01) with the TCLP-Cd, -Pb, and -Zn concentrations; shoot Cd, Pb, and Zn concentrations; and their translocation from roots to shoots (Table 3), confirming that soil pH is a key factor influencing plant growth and controlling heavy metal bioavailability and accumulation by plants, at least in the acidic soil we aimed to remedy. Furthermore, the liming effect also facilitates the precipitation of heavy metals by forming insoluble oxyhydroxides or hydroxides [47].

Due to its chemical composition, HAP can release OH^-^ to enhance soil pH, hence inducing the liming effect [10,11]. In addition, the pH values of OM and BC in our present study were 8.58 and 9.64, respectively; thus, they induced liming effects. OM has been shown to increase soil pH, particularly in acidic soils [53,54], which could be largely due to the addition of basic cations and production of NH_3_ during manure decomposition [55]. Manures contain some organic acid anions, which will consume protons during their decomposition, leading to an increase in soil pH [53]. BC is normally alkaline due to its high alkaline mineral content and could thus increase soil pH with increasing application rates, particularly in acid soils [15,30]. In conclusion, the liming effect is one of the common mechanisms accounting for heavy metal remediation by the three amendments.

More interestingly, in multiple-metal-contaminated soils, the presence of one metal can alleviate the immobilization efficiency of the other due to the competition among them for adsorption sites [47]. In our present study, Zn was more difficult to stabilize with HAP compared to Pb and Cd (Figure 2), confirming that Zn is a rather mobile element and easily out-competed by other divalent metals [56]. Similar results were also observed in a previous study: the presence of phosphate greatly decreased the concentrations of metals in solution, particularly Pb and Cd, but slightly reduced Zn concentrations [57]. However, BC was only effective in the stabilization of Zn (Figure 2), indicating its different immobilization mechanisms compared to HAP. As a consequence, the co-application of various amendments may be more feasible for the remediation of multiple-metal-contaminated soils [58,59].

The TF reflects the capacity of plants to transfer heavy metals from roots to aboveground parts. High levels of plant P may interfere with the translocation of Zn from roots to aerial parts [60]. In our present study, HAP was the most effective amendment in reducing the TF of Zn, as well as Cd and Pb, suggesting that the improved plant P can also depress the translocation of Cd and Pb from roots to shoots. A low TF means less accumulation of contaminants in leaves and seeds; thereby, HAP may be an ideal amendment for the cleaner production of food crops.

Sufficient nutrients are essential for plants’ survival in contaminated soils. Our present results show the three amendments have different nutritional functions. The solubility of the inorganic P fertilizer HAP is relatively low at a higher pH, but increases rapidly with a decreasing soil pH < 6 [61]. Since the soil we used is acidic with a low pH of 5, HAP has a high phytoavailability. HAP not only improved plant P nutrition as expected, but also increased plant K, Fe, and Cu uptake (Figure 6 and Figure 7), indicating that it can provide P directly and also improve other nutrients indirectly. OM is a slow-release fertilizer with low available nutrients, which explains why only 1% OM improved plant nutrition and growth but 0.1% OM did not. Comparatively, due to its low fertility, BC did not improve plant mineral nutrition and growth. BC contains macro- and micro-nutrients, organic matter, and ash and thus can improve plant nutrition [14]. However, due to its excellent adsorption capacity and abundant functional groups, BC sometimes precipitates nutrients into forms with a low bioavailability [14]. Thus, BC does not always necessarily improve plant nutrition.

Another interesting finding is that high doses (1%) of the three amendments, especially HAP, decreased plant Mn uptake but increased Fe and Cu uptake (Figure 7). The bioavailability and uptake of Mn are influenced by many factors such as soil pH, organic matter, redox potential, and other nutrients [60]. Indeed, the DTPA-Mn concentrations in the soil after plant harvest were most significantly decreased by amendments compared to Fe and Cu (Figure S2). Generally, Mn uptake by plants is more dependent on soil pH compared to the uptake of other micronutrients. The increased soil pH can partly explain the lower Mn uptake induced by the amendments. However, this is not the sole reason, because 0.1% HAP also increased the soil pH but did not significantly change the plant Mn uptake. Previous studies have found that high soil and plant P generally depresses plant Mn uptake. For example, the application of triple superphosphate resulted in elevated plant P, which interfered with the uptake and translocation of Mn, leading to decreased leaf Mn concentrations [62]. Similar to our findings, Boisson et al. [63] observed lower Mn uptake and symptoms of Mn deficiency in the leaves of maize plants grown in heavy metal-contaminated soil with a high HAP application rate. In addition, the Mn^2+^ ion has similar properties to alkaline cations such as Ca^2+^, and there may be an antagonistic relationship between Mn^2+^ and Ca^2+^ absorption [64]. Besides HAP, both OM and BC contain abundant Ca, which may compete with Mn and thus contribute to the decreased plant Mn uptake. Another explanation may be attributed to Fe–Mn antagonism in higher plants [65]. High doses of amendments did not inhibit or even enhanced Fe uptake by plants (Figure 7b), leading to higher Fe/Mn ratios, which may decrease Mn uptake and translocation from roots to shoots. These findings suggest that (1) these amendments may have great potential in reducing Mn toxicity and remediating Mn-contaminated soil, and (2) the application of the amendments may cause a plant deficiency of Mn, which should be supplemented for better plant growth in amended soils.

Lastly, our results show significant dose-dependent effects and type–dose interactions for most of the parameters measured (Table 2). For example, HAP and OM at 1% doses caused lower plant Cd, Pb, and Zn concentrations but higher plant biomass compared to the 0.1% doses. Logically, higher dose of amendments can provide more active reaction sites for the adsorption, precipitation, and complexation of metals, and more beneficial nutrients for plants. However, the high-dose application of amendments may bring high costs and environmental side-effects. Low-cost, environmentally friendly amendments need to be developed for future soil remediation programs.

## 5. Conclusions

Here, we compared the effects of HAP, OM, and BC on plant growth, heavy metal accumulation by maize, and heavy metal immobilization in an acidic soil contaminated with Cd, Pb, and Zn, and their potential for safe crop production. The three amendments played roles in improving plant growth and nutrition, reducing heavy metal uptake and translocation from roots to shoots, and increasing soil pH, but the effects were dependent on the amendment type and dose. Overall, HAP produced the most pronounced effect. When applied at the 1% dose, HAP increased the shoot and root biomass by 188% and 157%, respectively, and reduced the shoot Cd, Pb, and Zn concentrations by 67%, 85%, and 84%, respectively. Plant P, K, Fe, and Cu nutrition and soil pH were significantly improved. The three amendments decreased the bioavailability of at least one heavy metal, and HAP showed the most effective immobilization effects, which can be ascribed to its liming effects and the formation of sparingly soluble Cd-, Pb-, and Zn-P-containing minerals. In conclusion, among the three amendments, HAP was the optimal amendment for stabilizing heavy metals, reducing heavy metal uptake, and promoting plant growth, suggesting a promising potential for safe crop production in contaminated soil.

## Figures and Tables

**Table 1:** Physical and chemical properties of the test soil.

Item	Value	Screening Value *	Intervention Value *
pH (soil/water, 1:2.5, *w*/*v*)	5.0		
Total Cd	2.6 mg/kg	0.3	1.5
Total Pb	1796 mg/kg	70	400
Total Zn	1603 mg/kg	200	
TCLP-Cd	2.25 mg/kg		
TCLP-Pb	136.8 mg/kg		
TCLP-Zn	371.6 mg/kg		
Organic matter	25.8 g/kg		
Olsen P	27 mg/kg		
NH_4_OAc extractable K	26.3 mg/kg		
Alkali-hydrolyzable N	118 mg/kg		
DTPA-Fe	154.6 mg/kg		
DTPA-Mn	68.5 mg/kg		
DTPA-Cu	7.5 mg/kg		
Cation exchange capacity	3.15 cmol/kg		
Soil type	Paddy soil		

* Data are from the “Soil Environmental Quality—Risk control standard for soil contamination of agricultural land (GB 15618-2018)”. “Screening value” means potential risks for agricultural food security, crop growth, or soil quality if pollutant concentrations exceed this value. “Intervention value” means the soil should be strictly controlled for agricultural production if pollutant concentrations exceed this value.

**Table 2:** Significance levels (*F* value) of amendment type, amendment dose, and their interactions for measured variables according to a two-way ANOVA analysis.

Variables	Amendment Type (T)	Amendment Dose (D)	T × D
Soil pH	151.966 ***	221.228 ***	28.943 ***
TCLP-Cd	218.36 ***	165.035 ***	31.889 ***
TCLP-Pb	226.041 ***	160.43 ***	65.231 ***
TCLP-Zn	6.124 **	22.046 ***	0.991 ns
Shoot dry weight	165.465 ***	83.228 ***	30.445 ***
Root dry weight	57.247 ***	12.865 ***	5.523 **
Shoot Cd conc.	132.490 ***	31.075 ***	13.495 ***
Shoot Pb conc.	115.236 ***	57.883 ***	13.821 ***
Shoot Zn conc.	101.379 ***	200.338 ***	24.518 ***
Root Cd conc.	24.638 ***	0.991 ns	4.837 *
Root Pb conc.	20.984 ***	26.844 ***	0.800ns
Root Zn conc.	153.407 ***	106.085 ***	40.609 ***
TF of Cd	7.970 **	5.010 *	1.748 ns
TF of Pb	30.988 ***	1.841 ns	14.799 ***
TF of Zn	5.571 **	26.472 ***	4.810 *
Shoot Cd uptake	11.673 ***	2.770 ns	13.775 ***
Shoot Pb uptake	19.292 ***	13.781 ***	6.631 **
Shoot Zn uptake	1.625 ns	50.150 ***	14.606 ***
Root Cd uptake	4.499 *	1.321 ns	2.713 ns
Root Pb uptake	1.246 ns	3.628 ns	0.404 ns
Root Zn uptake	4.402 *	17.688 ***	5.024 *

* *p* < 0.05, ** *p* < 0.01, *** *p* < 0.001, ns non-significance.

**Table 3:** Pearson coefficients of correlation between some soil and plant traits.

Item	Soil pH	Dry Weight		Cd conc.		Pb conc.		Zn conc.		TF		
		Shoot	Root	Shoot	Root	Shoot	Root	Shoot	Root	Cd	Pb	Zn
Soil pH		0.934 **	0.804 **	−0.877 **	−0.687 **	−0.928 **	−0.831 **	−0.956 **	−0.941 **	−0.624 **	−0.727 **	−0.487 **
TCLP-Cd	−0.936 **	−0.955 **	−0.877 **	0.907 **	0.717 **					0.679 **		
TCLP-Pb	−0.939 **	−0.937 **	−0.834 **			0.917 **	0.791 **				0.794 **	
TCLP-Zn	−0.765 **	−0.696 **	−0.565 **					0.746 **	0.724 **			0.250 ns

** *p* < 0.01; ns, non-significance.

## Supplementary Materials

The following are available online at https://www.mdpi.com/2305-6304/8/4/102/s1, Table S1: Significance levels (*p* values) of amendment type, amendment dose, and their interactions for measured variables (P, K, Fe, Mn, and Cu) according to a two-way ANOVA analysis, Figure S1: SEM images (upper) and FTIR spectra (lower) of biochar, Figure S2: DTPA-Fe, -Mn, and -Cu concentrations (means ± SD, *n* = 6) in soil after plant harvest. CK represents the control treatment. Different letters above or below the bars indicate significant differences among all means according to a one-way ANOVA followed by Duncan’s test (*p* < 0.05). Two-way ANOVA results are shown in Table S1.
