# Supplementary material for: Effects of Soil Amendments on Heavy Metal Immobilization and Accumulation by Maize Grown in a Multiple-Metal-Contaminated Soil and Their Potential for Safe Crop Production
Source: Toxics. 2020 Nov 11;8(4):102. doi: 10.3390/toxics8040102 (PMC7712267; doi:10.3390/toxics8040102)
Supplement: Supplementary file 1 [file toxics-08-00102-s001.pdf]

Supplementary Materials: Effects of Soil Amendments on Heavy Metal Immobilization and Accumulation by Maize Grown in a Multiple-Metal-Contaminated Soil and Their Potential for Safe Crop Production

Fayuan Wang, Shuqi Zhang, Peng Cheng, Shuwu Zhang and Yuhuan Sun

Table S1. Significance levels (*p* value) of amendment type, amendment dose, and their interactions on measured variables (P, K, Fe, Mn and Cu) by a two-way ANOVA analysis.

Variables	Amendment Type (T)	Amendment Dose (D)	T × D
Shoot P conc.	236.411***	243.775***	202.714***
Shoot K conc.	24.744***	71.823***	7.011**
Shoot Fe conc.	58.225***	25.385***	14.866***
Shoot Mn conc.	88.976***	93.896***	11.498***
Shoot Cu conc.	11.576***	1.028ns	40.076***
Root P conc.	295.117***	207.056***	156.239***
Root K conc.	235.640***	172.058***	103.043***
Root Fe conc.	23.907***	20.499***	2.597ns
Root Mn conc.	96.501***	217.890***	33.797***
Root Cu conc.	7.805**	2.005ns	16.195***
Shoot P uptake	307.734***	247.462***	167.669***
Shoot K uptake	249.008***	242.340***	80.599***
Shoot Fe uptake	34.682***	2.218ns	8.664***
Shoot Mn uptake	18.172***	48.152***	8.585***
Shoot Cu uptake	80.600***	41.690***	14.540***
Root P uptake	250.774***	136.885***	91.869***
Root K uptake	171.234***	114.772***	87.856***
Root Fe uptake	72.347***	34.057***	10.710***
Root Mn uptake	13.804***	75.914***	6.202**
Root Cu uptake	39.007***	10.583**	4.835*
DTPA-Cd conc.	178.444***	235.095***	60.496***
DTPA-Pb conc.	29.226***	8.784**	25.119***
DTPA-Zn conc.	1.145ns	26.083***	13.626***

*$p < 0.05$, **$p < 0.01$, ***$p < 0.001$, ns non-significance.

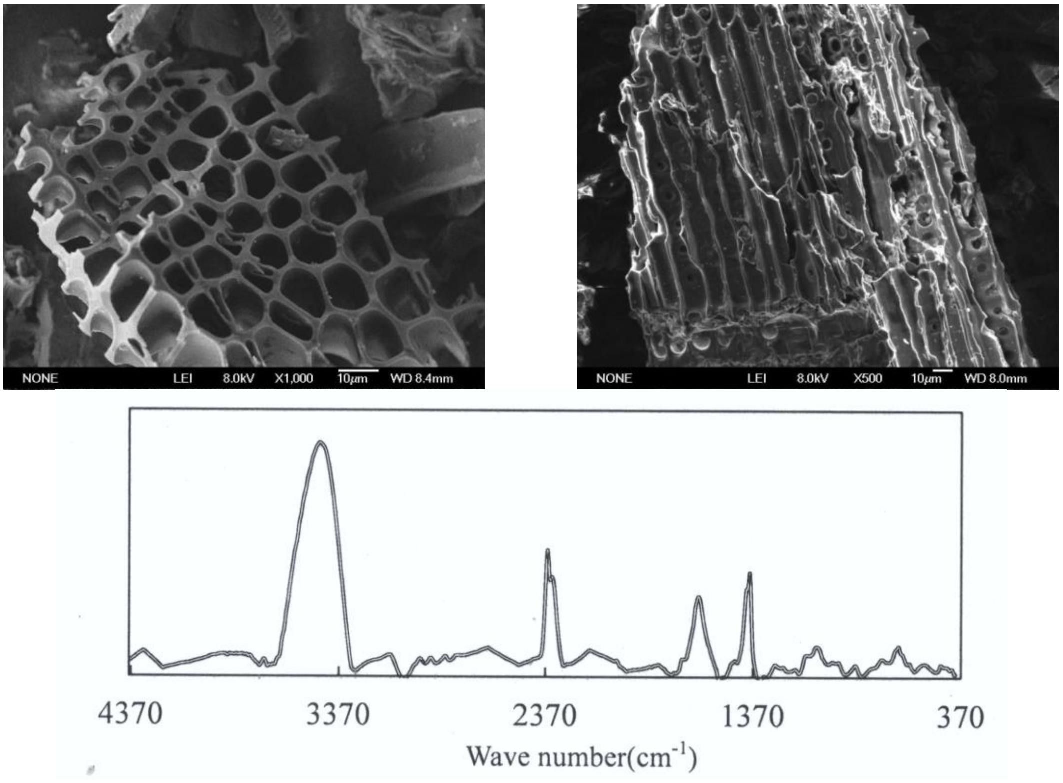

Figure S1. SEM images (upper) and FTIR spectra (lower) of biochar.

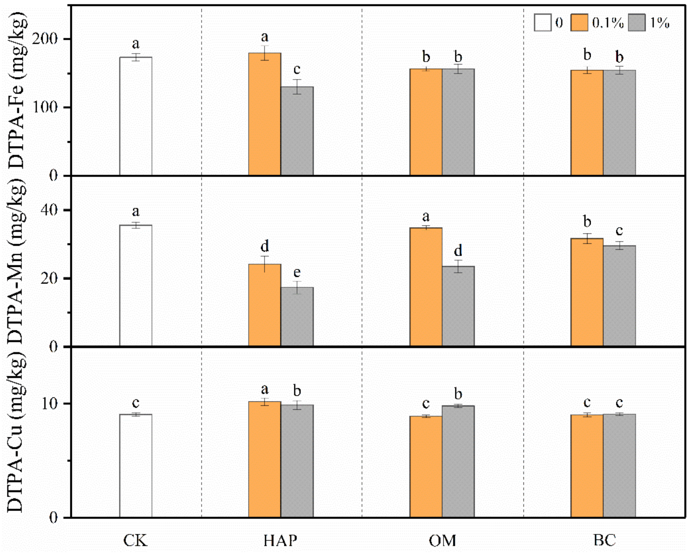

Figure S2. DTPA-Fe, -Mn and -Cu concentrations (means ± SD, $n = 6$) in soil after plant harvest. CK represents the control treatment. Different letters above or below the bars indicate significant differences among all means using a one-way ANOVA followed by Duncan's test ($p < 0.05$). Two-way ANOVA results are shown in Table S1.
